# Evodiamine Eliminates Colon Cancer Stem Cells via Suppressing Notch and Wnt Signaling

**DOI:** 10.3390/molecules24244520

**Published:** 2019-12-10

**Authors:** Hyejin Kim, Yeongji Yu, SeokGyeong Choi, Hani Lee, Jinsuh Yu, Jeong-Ho Lee, Woo-Young Kim

**Affiliations:** 1 College of Pharmacy, Sookmyung Women’s University, Cheongparo-47 Gil, Yongsan Gu, Seoul 04312, Korea; hyejin9133@gmail.com (H.K.); djmd3353@naver.com (Y.Y.); choi9174@gmail.com (S.C.); lhani0123@naver.com (H.L.); Jinsuhyu310@gmail.com (J.Y.); 2 Inland Aquaculture Research Center, National Institute of Fisheries Science, Changwon 51688, Korea; jhlee7124@korea.kr; 3 Research Institute of Pharmaceutical Sciences, Sookmyung Women’s University, Cheongparo-47 Gil, Yongsan Gu, Seoul 04312, Korea

**Keywords:** evodiamine, colorectal cancer, cancer stem cell, notch, WNT

## Abstract

Evodiamine, an alkaloid contained in traditional Asian herbal medicines that have been used for hundreds years, is interesting due to its cytotoxic effects against many cancers. We examined the effect of evodiamine on the cancer stem cell (CSC) population and the bulk cultured cancer cells (BCC) of colon cancers to examine the double targeting effect. We found that three colon cancer cell lines’ BCC and CSC are effectively targeted by evodiamine. Evodiamine was able to suppress BCC proliferation and induce apoptosis of the cells captured in G2/M phase, as previously reported. However, evodiamine did not cause the accumulation of CSCs at a certain stage of the cell cycle, resulting in the elimination of stemness through an unknown mechanism. By analyzing the expression of 84 genes related to CSCs in two colon cancer cell lines’ CSC, as well as performing further informatics analyses, and quantitative RT-PCR analyses of 24 CSC genes, we found that evodiamine suppressed the expression of the genes that control key signaling pathways of CSC, namely, WNT and NOTCH signaling, to lead CSC elimination. These results suggest that evodiamine should be further developed for targeting both BCCs and CSCs in colon cancers.

## 1. Introduction

Over the last half century, intense efforts led by developed countries have been devoted to cancer research, many new anticancer therapeutic strategies were developed, and there have been significant improvements in the prognosis of several subtypes of cancer. However, the mortality of most cancer types is still very high, leaving the disease one of top causes of death world wide [1]. At least some of the reason may be that most of those drugs target rapidly-growing cells (named bulk cancer cells, BCC), making it is very difficult to avoid cytotoxic side effects to rapidly-growing normal cells [2,3]. Limiting the concentration of the cancer drugs may result in the survival of residual cancer cells in the primary or secondary tissues. As a consequence, recurrence and metastasis following the primary responses make the cancer patients less likely to survive.

A small population in the tumors, “cancer stem cells or CSCs”, is also believed to have pivotal roles in cancer initiation, resistance and metastasis [4]. Ironically, many therapeutic approaches, including radiation and chemotherapeutic agents, may even induce CSC number and the potentials [5,6,7]. This CSC population may share many normal stem cell-like characteristics, and therefore, it is difficult to eliminate with the BCC-targeting strategies. Accordingly, numerous attempts to selectively target this small population have been made. CSCs also share many signaling networks for their survival or “stemness” with the tissue stem cells, which include the Notch and WNT signaling pathways. Therefore, the inhibitors of these signaling have been developed and are being applied for clinical evaluation to eliminate not only the BCCs but also the CSCs [5]. However, the clinically approved CSC targeting agents are very limited so far [4,8], and the dual targeting strategy is not available yet.

To find druggable pathways that target CSCs and BCCs together, we performed siRNA screening and revealed several potential targets [9]. Several metabolic pathways may act as vulnerable selective CSC targets were identified. We also found common targets for CSC and BCC, and they are primarily related to basic cell functions, such as transcription and protein metabolism. Therefore, targeting these pathways may lead to greater toxicity to normal cells. If we could identify therapeutic candidates that can be applied to the BCC and the CSC from the herbal medicines used in Asian countries for thousands years, it would widen the chance to cure this fearful disease.

Colorectal cancer is the 3rd most common cancer type worldwide, and the incidence is believed to be related to diet. The mortality is very high due to late diagnosis resulting in metastasis and resistance to conventional therapies.

Evodiamine is a main component of plant *Evodiae fructus*, which has been used for long time in Asian medicine for symptoms including digestive problems [10]. The anti-proliferative, pro-apoptotic and anti-invasive effect of evodiamine on cancer cells has been reported by several studies [11,12]. However, those studies focused on the fast-growing cancer cells, BCCs. Recently, we found that, in comparable concentrations, this compound modulates the p53-p21-Rb pathways only in breast CSCs but not in BCCs holding them at G1 stage [13], and we hypothesized that evodiamine may also target the colon cancer cells.

We tested the potential for evodiamine as a colon CSC-targeting agent and compared the effect in BCCs to determine if the compound can be used to eliminate both populations of colon cancer cells. Evodiamine was an effect targeting agents for both BCC and CSC inducing apoptotic cell deaths and reduced the stem cell potential in vitro and in vivo. Biochemical and genetic mechanistic study revealed WNT and Notch pathway, which are essential for cancer stem cell signaling, are primarily modulated by evodiamine in CSC of two cell lines suggesting evodiamine as a strong candidate to target colorectal BCCs and CSCs together.

## 2. Results

**Figure 1:**
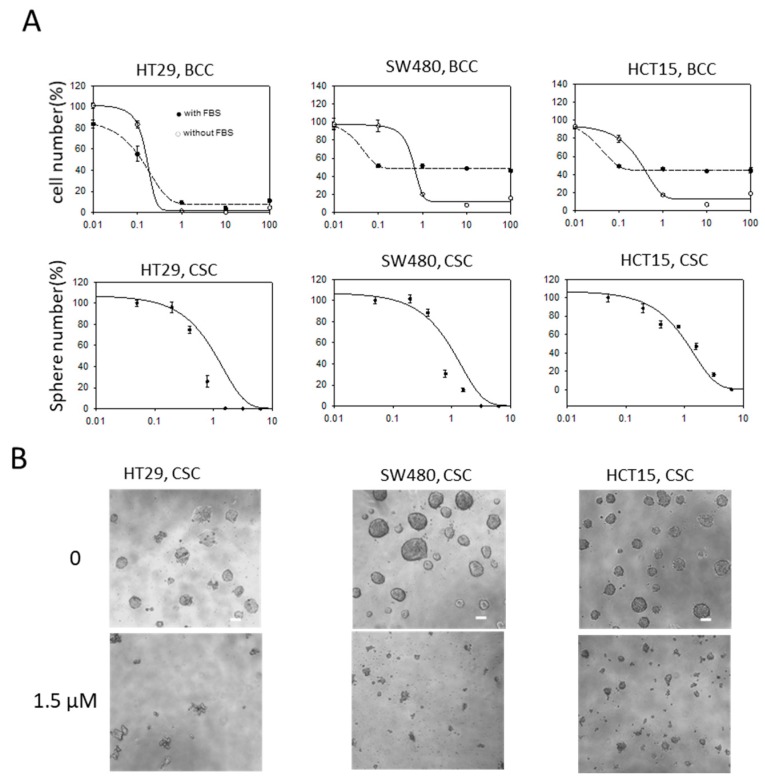
Anti-proliferative/survival activity of evodiamine on bulk cultured cancer cells (BCC) and cancer stem cells (CSC) of colon cancer cell lines. (**A**) Relative cell numbers or CSC sphere (bigger than 100 μm in diameter) numbers after treated with evodiamine for 3 days (BCC) or 5 days (CSC). Average of six independent experiments were shown with error of means. BCC were maintained with 10% FBS or without FBS. X axis, evodiamine (μM); (**B**) Representative images of CSC spheres under microscope (scale bar represents 100 μm).

To examine if evodiamine could be used to treat the colon BCCs and CSCs together, we tested if evodiamine suppressed the growth of both the BCCs and CSCs of colon cancers using three colon cancer cell lines (HT29, SW480, and HCT15). In BCC culture, the cells were maintained in the medium supplemented with FBS or without serum and attached on the culture dish. The cell growth of all three cell lines in both the BCC and CSC conditions was effectively inhibited by treatment with evodiamine at concentrations between 0.1 and 1 μM. At the low concentration, the serum-free condition cultured cells were more resistant than the FBS-supplemented cultured cells, but this resistance was reversed at higher concentrations in the SW480 and HCT15 cells. The growth of CSC spheres was also effectively inhibited at concentrations lower than 1 μM in all three tested cell lines (Figure 1A). The microscopic images of the CSCs showed almost no healthy CSC spheres at 1.5 μM evodiamine (Figure 1B).

**Figure 2:**
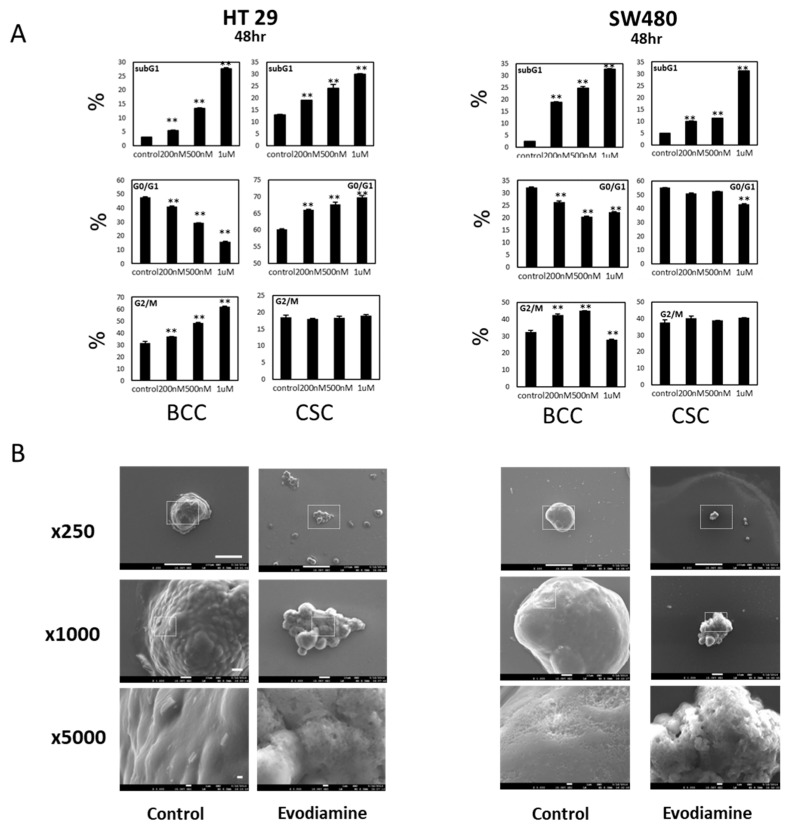
Cell cycle changed and cell death by evodiamine on BCC and CSC cells. (**A**) Cell cycle changes after evodiamine treatment for 48 h. G2/M accumulation in BCC is evident while no specific cell cycle blockage is seen in CSC but both induce cell death by evodiamine in a dose dependent manner. Average of four independent experiments were shown with error of means. (**B**) Scanning electron microscopy of CSCs showing apoptotic membranes in evodiamine treated CSC. Scale bar represents 100 μm, 10 μm, and 1 μm in 250×, 1000×, and 5000× images, respectively. ** *p* < 0.01 vs. control.

To examine whether the inhibitory effect on BCCs and CSCs occurs through G2/M arrest, as previously reported, we performed cell cycle analyses on two cell lines, HT29 and SW480. In both conditions, the dying population increased at 48 h of treatment in a dose-dependent manner (Figure 2A), and the dying fraction further increased at 72 h of treatment (Figures S1 and S2). However, the pattern of cell cycle changes induced by evodiamine was not evident in CSCs, while the decrease in G1 and the increase of G2/M were clearly detected in the BCCs of both cell lines. Since the SW480 cells at G2/M stages did not increase in a dose dependent way, probably due to the massive cell death accompanied, we checked the early responses at 16 h after treatment. At this time point the cells are accumulated at the G2/M phase in the dose dependent manner without massive cell death increase (Figure S3). Though the sub-G1 cells with fractionated nuclei and western blotting (Figure S4) strongly suggest that they undergo apoptosis we also observed those cells under a scanning electron microscope (SEM) to see the characteristics of apoptotic morphologies. The SEM images of CSCs of two cell lines treated with evodiamine showed typical apoptotic bodies [14] on the sphere surfaces [15] (Figure 2B). Therefore, we concluded that evodiamine did induce cell death in both colon BCCs and CSCs but acted via different mechanisms in these two cell populations.

**Figure 3:**
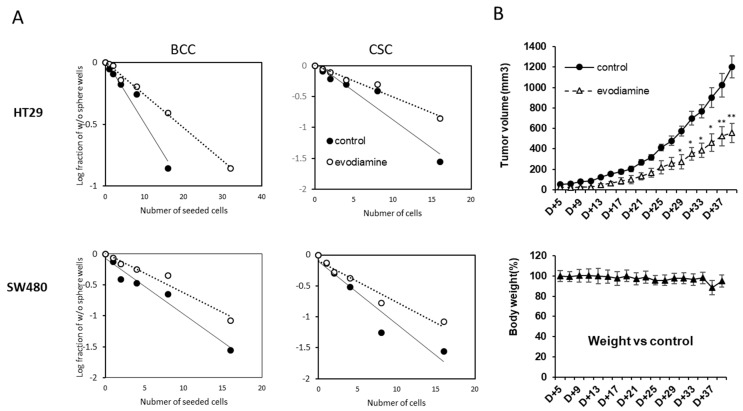
Evodiamine eliminated the cells with cancer stem cell activity in vitro and in vivo. (**A**) Limited dilution assay of the cells treated with evodiamine (200 nM for 24 h). (**B**) In vivo tumor growth activity of survived BCC cells after pretreated with evodiamine (1 μM for 24 h). *n* = 5 for each. Average with SEM. * *p* < 0.05; ** *p* < 0.01 vs. control.

Self-renewal activity is a surrogate marker for colon cancer CSC potential, and the population with this activity can be quantified using limiting dilution analysis (LDA) [16]. We tested if the short treatment of evodiamine indeed suppresses this self-renewal activity. Only the surviving cells after evodiamine treated (24 h) on the BCC culture condition were plated in 96-well plates using serial dilutions and in conditions for CSC sphere enrichment. The sphere formation ability decreased in the both cell lines, HT-29 and SW480 (Figure 3A left). The stem cell potential of the surviving evodiamine-treated cells in enriched CSCs also tested in the same manner. The self-renewal of the surviving cells was decreased by evodiamine (Figure 3A right). We also used a more direct marker of CSC potential, the tumor formation activity in vivo. The same number of short-evodiamine treated cells and control cells were subcutaneously implanted in SCID mice and showed different tumor growth in vivo. These results collectively suggest that the cells have CSC potentials may be eliminated by evodiamine in BCCs and CSCs in culture (Figure 3B).

**Figure 4:**
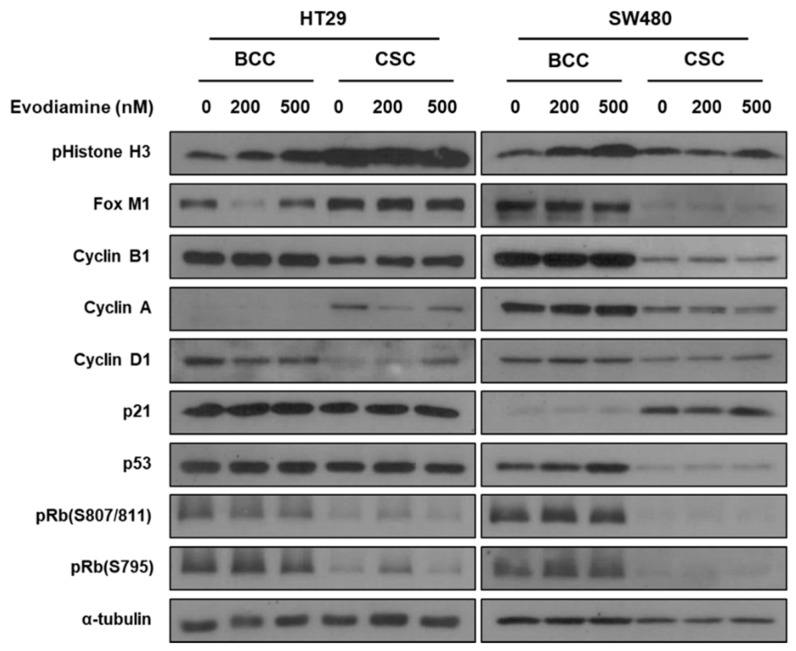
Changes of cellular signaling by evodiamine in BCC and CSC. The cells treated with Evodiamine (200 nM and 500 nM) were treated to the bulk cultured or CSC enriched cells for 24 h and the cell lysates were applied to western blotting.

The different patterns of cell cycle changes in the CSCs and BCCs suggest the molecular mechanism through which evodiamine induces cell death in the two cell types may be independent of each other. The BCCs were captured at G2/M checkpoint and may undergo apoptosis as we published in breast cancer [13]. However, the CSCs did not show arrest at a specific checkpoint unlikely from the effect in breast cancer, suggesting novel pathways may regulate them. We first tested if previously reported p53-p21-Rb pathway [13], in both cell lines and in both conditions are changed by evodiamine (Figure 4). In both cell lines, evodiamine increased phospho histone 3 (M phase marker) in BCCs only, as described before [13], and similar results were found with the cell cycle analyses (Figure 2A). However, interestingly, the p53-p21-Rb pathway, which has been reported by our group to be modulated in breast CSCs to hold them in G1 [13], was not changed in the tested colon CSCs (levels of p53, p21, and pRbs did not increase as in breast cancer cells). Another M phase marker PLK-1 also increase in SW480 (Figure S4). Interestingly the Cyclin B1 did not change in the BCC condition. The oscillation of FOXM1 and Cyclin A expression was unique in HT-29. These data and the cell cycle data strongly show that the dual targeting of BCCs and CSCs by evodiamine is not mediated by a common mechanism in these colon cancer cell lines. In addition, the effect on CSCs occurs through an independent mechanism from that on BCCs, and it is also different from that on breast CSCs.

**Figure 5:**
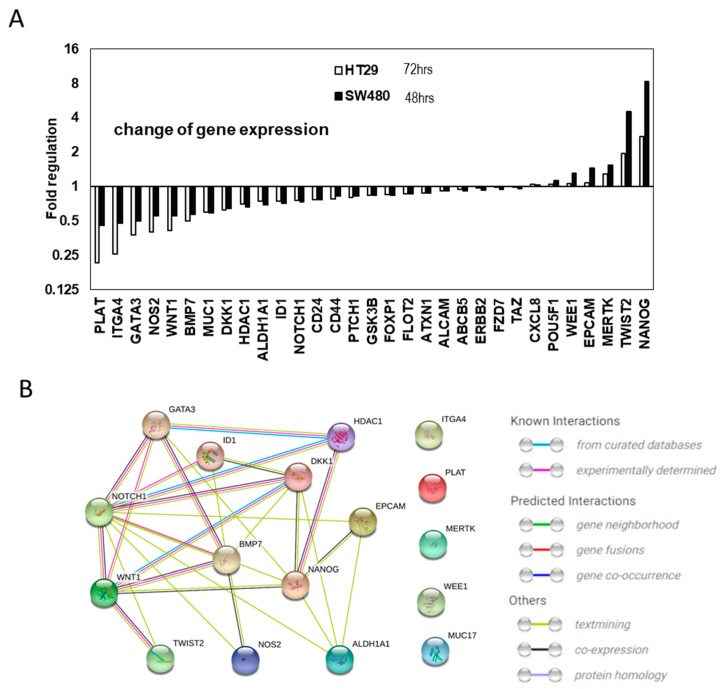
Cancer stem cell gene expression changes by evodiamine. (**A**) Cancer stem cells gene expression changes common between 48 h treated SW480 and 72 h treated HT29 CSC with mild (200 nM) evodiamine treatment. (**B**) The interaction of the commonly changes 17 genes is focused to NOTCH1 and WNT1.

To identify the molecular mechanism through which evodiamine suppresses CSC enrichment, we employed small-scale screening using a quantitative RT-PCR array (RT^2^ profile PCR array, Qiagen). The changes in the expression in 84 genes related to CSC biology were screening together (Figure 5). 

We treated CSCs with 200 nM evodiamine, which did not significantly kill the CSC spheres at 5 days of treatment (Figure 1A), to identify the primary targets modulated by evodiamine. After 48 h of treatment of SW480 and 72 h of HT29, in the CSC enrichment conditions, there were changes in the expression of several genes. The changes in these two conditions were analyzed and are shown in Figure S5. Many genes related to CSCs changed in the two different cell lines treated for different time periods. Some groups of genes commonly changed, whereas others showed different patterns of changes. These unique patterns may suggest that unique cellular molecular networks exist in these two cell lines, or the difference may be attributable to different lengths of treatment (Figure S5).

To determine the common mechanism targeting these two cell lines’ CSCs, we further analyzed the data and the selected genes for which expression was commonly increased and decreased after this mild treatment of evodiamine in both the HT29 and SW480 cells lines (Figure 5A). Since the cell cycle results suggested that SW480 died about 1 day earlier than HT 29 did after evodiamine treatment, we checked the gene expression at 48 h and 72 h after treatment respectively. As seen in the graph, all commonly diminished genes decreased to a lesser extent after 48 h of treatment than after 72 h. Of the commonly decreased or increased genes, the changes of more than 25% (17 genes) were analyzed for their interaction using the String database [17]. Of those genes, seven genes (*HDAC1, DKK1, GATA3, ID1, BMP7, WNT1*, and *NANOG*) have been experimentally demonstrated to interact with *NOTCH1* and five genes (*ID1, BMP7, TWIST2, DKK1,* and *NOTCH1*) that interact with WNT1 were identified. In addition, six genes (*NANOG, ALDH1, MUC1, NOS2, EPCAM,* and *TWIST*) for NOTCH1 and one gene (*NANOG*) for WNT1 were expected to functionally interact. Notch and WNT signaling are the pivotal players of tissue stem cells and cancer stem cells [18,19,20,21,22] for their stem cell potential and proliferation. In colon stem cells, Notch and WNT signaling is the most important signaling for asymmetric and symmetric division-mediated self-renewal, and these signaling pathways also interact with each other in cancer [22,23,24,25,26,27].

**Figure 6:**
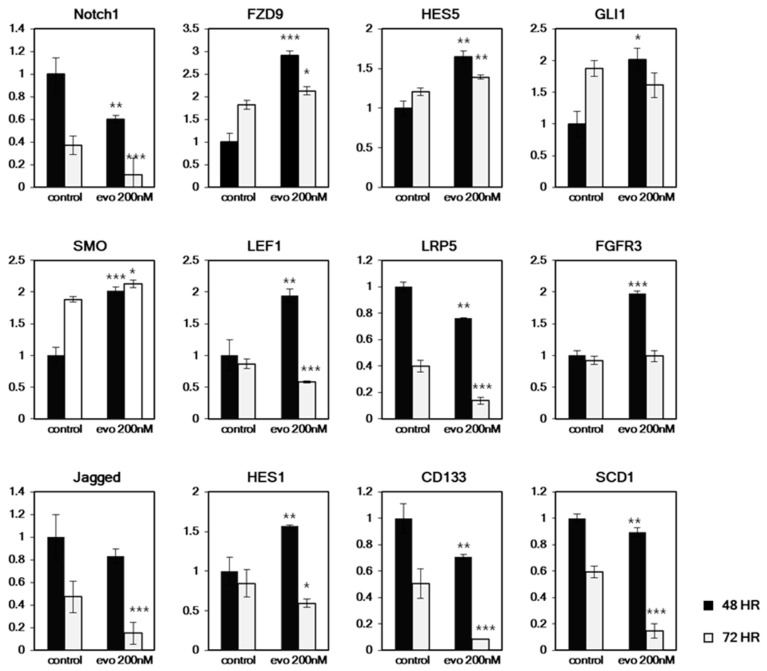
Time dependent changes of CSC related cell signaling genes and marker genes expression in HT29. The CSC enriched cultured cells were treated with evodiamine for 48 h or 72 h and the mRNA expressions were tested with quantitative RT-PCR. Only Notch1, LRP5, Jagged, CD133 and SCD1 showed similar expression changes in time dependent manner. * *p* < 0.05; ** *p* < 0.01; *** *p* < 0.001 vs. each controls.

Based on this RT^-^ PCR array result, we hypothesized that evodiamine suppresses NOTCH and WNT signaling. To test this hypothesis, we further analyzed time dependent changes of gene expression, related to main CSC signaling pathways. A mild concentration of evodiamine (200 nM) was treated to HT29-derived CSCs for 48 h and 72 h, and the expression of NOTCH signaling (*Notch1, HES5, HES1*, and *Jagged*) and WNT signaling (*LRP5* and *LEF1*) genes was assessed. In addition, other genes related to FGF (*FGFR3*) signaling and Hh (*SMO, GLI*, and *FZD9*) with CSC markers (CD133 and SCD1) were also tested (Figure 6). Though we observed only limited inhibition of CSC sphere formation after 5 days treatment of 200 nM evodiamine (Figure 1), most of the gene expression exhibited changes with this low concentration. Interestingly, most of these genes show equivocal expression changes, increased and decreased/unchanged at 48 h and 72 h respectively. The induction of *HES5, HES1, FGFR3, SMO, GLI1*, and *LEF1* was observed at 48 h, but these genes were decreased or unchanged at 72 h. Only *Notch1, Jagged, LRP5, CD133,* and *SCD1* showed decreased expression at 48 h and were further suppressed by evodiamine at 72 h. Notch1 and Jagged are the receptor and ligand of Notch signaling, respectively, and Lrp5 is a coreceptor of WNT signaling. These results are consistent with the RT-PCR array analyses described in Figure 5. CD133 is a CSC marker of many cancer CSCs. In addition, SCD1 was also recently reported to be a CSC marker of ovarian [28] and colon [2] cancers. Therefore, it may be due the loss of stem cell population that most of the signaling genes tested at 72 h are downregulated, while only a small number of genes are downregulated at 48 h when a CSC marker SCD1 expressing population decrease is not evident yet.

## 3. Discussion

Developing traditionally used herb medicine drugs as new therapeutic agents for cancer research may be attractive for several reasons based on long history of use. First, at least some (directly related to cancer or not) biological efficacy is already warranted by empirical evidence for hundreds years. Second, a range of dose for safety and toxicity can be estimated by accumulated knowledge’s. Herbs containing evodiamine have been used for a long time in East Asia for the treatment of a range of conditions, including digestive problems and pain [29]. Recently, an increasing number of reports have shown that this chemical has significant anti-proliferative and anti-invasive activity in several types of cancer [13,30,31,32]. We found and reported that evodiamine has selective or superior anti-proliferative activity to the CSCs [13] over the BCCs of breast cancer. These results led us to test if this compound may suppress the BCCs and CSCs of colon cancer. Unexpectedly, the inhibitory effects on the BCCs and CSCs are comparable, suggesting the potential use of this compound to eliminate BCCs and CSCs together in colon cancer patients. The CSC decrease was confirmed by the limited dilution assay in vitro and by the tumor forming assay in vivo. In addition, the expression of known CSC markers also decreased. We tested ALDH1 activity, a widely used marker for cancer stem cells for many tissues; however, colon BCCs already harbor strong ALDH1 activity, so this marker cannot be used as a reliable stem cell marker in colon cancer cell lines [33].

As reported in BCCs in many tissues and CSC in breast cancer, the CSCs of colon cancers died due to apoptosis at concentrations of evodiamine between 0.2 and 1 μM. Compared to the certain size (over 100 μm) of sphere numbers, which decreased very little in response to 200 nM evodiamine (Figure 1A), the CSC spheres seemed to successfully eliminate CSC marker expression (CD133, and SCD1 Figure 6) after 48 h of treatment. Interestingly, the genes changed by evodiamine in both SW480 treated for 48 h and HT29 treated for 72 h were related to two well-known stem cell key signaling pathways, Notch and WNT (Figure 5). Even though the cells in these two different conditions showed this common change, the group of genes modulated also have unique patterns (Figure S3). Notch signaling is a regulator of stem cell asymmetric division and is also essential for CSC maintenance. The suppression of Notch signaling in one daughter cell is essential for the asymmetric division and the designation to a nonstem cell state in colon cancer [25]. The inhibition of Notch in stem cells results in both daughter cells differentiating into nonstem cells, leading to early consumption of stem cells in vivo. Therefore, the aberrant Notch regulation by evodiamine may promote the cells to cease acting as CSCs and this unbalance make the cells differentiate or die. In addition, the WNT ligand expression decrease may be working directly on the canonical WNT/β-catenin signaling, which is strongly associated with colon carcinogenesis though the mutations of APC and β-catenin. However, it is more plausible that the suppression of the noncanonical WNT pathway may have an important role in the CSC effect because the mutations found in the colon cancers (more than 80%) are generally associated with the ligand-independent activation of canonical WNT signaling, may contribute to the BCC effect. Interestingly WNT signaling is also known to induce numb and suppress NOTCH signaling [34], while Notch1 is also a target of Notch signaling. Therefore, it is expected that the molecular regulatory feedback between these two pro-CSC signaling pathways may be disrupted by evodiamine.

We finally tried to analyze the time-dependent changes of CSC molecules using RT-PCR in HT29 cells that were treated for 48 h and 72 h with 200 nM drug. At this concentration, we started to see the CD133 and SCD1 expression change, and this change was accompanied by a decrease in Notch1, Jagged, and LRP5. The expression of these genes was further decreased as CD133 and SCD1 decreased. However, all other genes were rather activated at 48 h. It seems this is due to the suppressed Notch and WNT signaling in the cells, may lead feedback effects to the suppressed CSC signaling. The following decrease of most other genes at 72 h may be related to the CSC specific elimination already started (considering CD133 and SCD1 at this points).

Collectively, we demonstrated that evodiamine had an interesting potential for simultaneous targeting colon CSCs and BCCs. The molecular mechanism of BCC targeting may be similar to the previously known mechanisms for other tissue cancers, which arrests the cells at the G2/M checkpoint and induces apoptosis. However, it primarily suppresses expression of WNT and NOTCH signaling genes, essential for CSC stemness or/and the maintenance. We therefore suggest that evodiamin’s interesting dual targeting effect might act through CSC-specific signaling in colon CSCs, which needs to be investigated further for development for clinical use.

## 4. Materials and Methods

### 4.1. Cell Culture

The human colon adenocarcinoma cell lines, HT29, HCT15, and SW480 were purchased from American Type Culture Collection (ATCC, Manassas, VA, USA). The cells were cultured in Dulbecco’s modified Eagle’s medium or RPMI1640 (both from Corning, Corning, NY, USA) supplemented with 10% fetal bovine serum (FBS, Gibco, USA) and 100 units/ml Anti/anti (Gibco). CSCs were enriched in DMEM/F-12 (Thermo Fisher Scientific, Waltham, MA, USA) supplemented with EGF (Gibco, 20 ng/mL) basic fibroblast growth factor (Gibco, 20 ng/mL), and B27 supplement (Gibco, 1X) on in house prepared ultra low adhesive culture plates, as described previously [35]. All cells were cultured at 37 °C in a humidified incubator with 5% CO_2_. All plastics culture wares were purchased from SPL (Korea). For cell counting, the cells in 96 wells were stained with Hoechst 33342 (Sigma) and imaged using a Cytation™ 3 (BioTek Instruments, Inc. Winooski, VT, USA). The number of CSC spheres with a diameter over 100 μm was counted after taking pictures with Nikon microscope. Evodiamine was purchased from Sigma (USA). The antibodies were acquired from Cell Signaling Technology (Danvers, MA, USA).

### 4.2. Quantitative Reverse Transcription PCR (qRT-PCR)

Total RNA was collected from CSCs and converted to cDNA using RT^2^ First Strand Kit (Qiagen, Venlo, Netherlands) and applied to RT^2^ Profiler™ PCR Array Human Cancer Stem Cells (PAHS-176, Qiagen) to examine 84 CSC related target genes together in Stepone Plus (ABI, USA). The data were further analyzed with Strings database (https://string-db.org/cgi/network.pl) [15].

Total RNA was also extracted from collected CSCs and BCCs with TRIsure (Bioline, Korea) and converted to cDNA with SuperScript II (Thermo Fisher Scientific). qRT-PCR was carried out [3,4] using SensiFAST SYBR Hi-ROX reagents (Bioline, Memphis, TN, USA). Ribosomal RNA L32 and actin were used as internal controls. The primers used are listed in Table S1.

### 4.3. Limited Dilution Assay

Limited dilution assay to determine self-renewal active cell density was performed based on previously published methods [16]. Briefly, evodiamine was treated to BCC and CSCs cultured for 24 h and the survived cells only were placed in low attachment 96 well plates with serial two fold dilutions. After 5 days, the percentage of wells without CSC spheres was calculated and plotted against the number of cells per wells.

### 4.4. Tumor Xenograft Assay

All experiments were pre-approved by IACUC of Sookmyung Women’s University. Alive BCC cells after treated with Evodiamine (1 μM) for 24 h were counted with trypan blue exclusion assay and 1 × 10^6^ alive cells were subcutaneously implanted in dorsal flank of in NOD/SCID (Koatech, Korea). The size of the tumor was measured with calipers, calculated by width × width × length × 1/2 every other days. The weight was also measured every other day. In each group, five mice were used. All mice were maintained in specific pathogen free facilities on a 12 hours light/12 hours dark cycle. At the endpoints, the mice are euthanized with excess CO_2_ gas.

### 4.5. Flow Cytometry

Dissociated single cells were treated with 250 μL 0.6% Triton X-100 containing 50 μg of RNaseA for 1 h at room temperature and stained by 5 μL of 1 mg/ml Propidium iodide. An assay was performed by Calibar (BD, Franklin Lakes, NJ, USA) and the data was analyzed by Flowjo software (V10, BD).

### 4.6. Scanning Electron Microscopy

Spheres were fixed using 4% formaldehyde for 20 min and washed with PBS. For dehydration, the samples were immersed in ethanol and displaced into hexamethyldisilazane (Sigma-Aldrich). For mounting the samples, the samples were sputter coated with gold on aluminum stubs with adhesive carbon tapes. The samples were observed by scanning electron microscope, JSM-7600F (Jeol, Akishima, Japan).

### 4.7. Western Blotting

The BCCs and CSCs were treated with evodiamine for specified time and the cells were harvested in the lysis buffer containing Tris-Cl pH 7.4, 1% NP-40, 1% triton X-100, 0.25% sodium deoxycholate, 150 mM sodium chloride, 10% glycerol, protease inhibitors (complete mini, Roche, South San Francisco, CA, USA), and phosphatase inhibitors (1 mM Na_3_VO_4_, 1 mM NaF, and 20 mM β-glycerophosphate) and sonicated. The cleared lysates were resolved in SDS-polyacrylamide gel by electrophoresis and then transferred to a PVDF membrane (Bio-rad, Hercules, CA, USA). The membranes were blocked by 5% non-fat dry milk in a buffer containing 20 mM Tris pH 7.4, 150 mM NaCl and 0.1 % Tween-20, then probed with primary antibodies and secondary antibodies with horse radish peroxidase. The signals were detected Supersignal West Femto (Thermo, Waltham, MA, USA), or Novex (Invitrogen, Grand Island, NY, USA). All original raw western blotting images are seen in Figure S6. The Abs used are listed in Table S2.

## 5. Conclusions

Evodiamine suppresses WNT and NOTCH signaling in CSC and can be used for dual targeting agents for BCC and CSC of colon cancer.

## Supplementary Materials

The following are available online. Figure S1. Cell cycle analyses of HT29. The cells cultured in bulk (BCC) and CSC enriched (CSC) are analyzed. Forty-eight hours and 72 h after treatment of evodiamine at the indicated concentration, the cell were applied to FACS analyses. Figure S2. Cell cycle analyses of SW480. The cells cultured in bulk (BCC) and CSC enriched (CSC) are analyzed. Eight hours and 72 h after treatment of evodiamine at the indicated concentration, the cell were applied to FACS analyses. Figure S3. Cell cycle analyses of SW480 at shorter treatment of evodiamine. The cells cultured in bulk (BCC) at 16 h showed clearer accumulation at G2/M phase. Figure S4. Changes of an M phase marker PLK1 and apoptotic marker PARP1 by evodiamine treatment. Figure S5. The gene expression changes of CSC regulating genes in HT29 and SW 480 after evodiamine treatment for 48 h and 72 h respectively. Figure S6a–c. Whole blot raw images for the western results. Table S1. RT-PCR primers used in this study. Table S2. Antibodies used in this study.
