# Supplementary material for: Evodiamine Eliminates Colon Cancer Stem Cells via Suppressing Notch and Wnt Signaling
Source: Molecules. 2019 Dec 10;24(24):4520. doi: 10.3390/molecules24244520 (PMC6943729; doi:10.3390/molecules24244520)
Supplement: Supplementary file 1 [file molecules-24-04520-s001.pdf]

Supplementary Figure 1

HT29

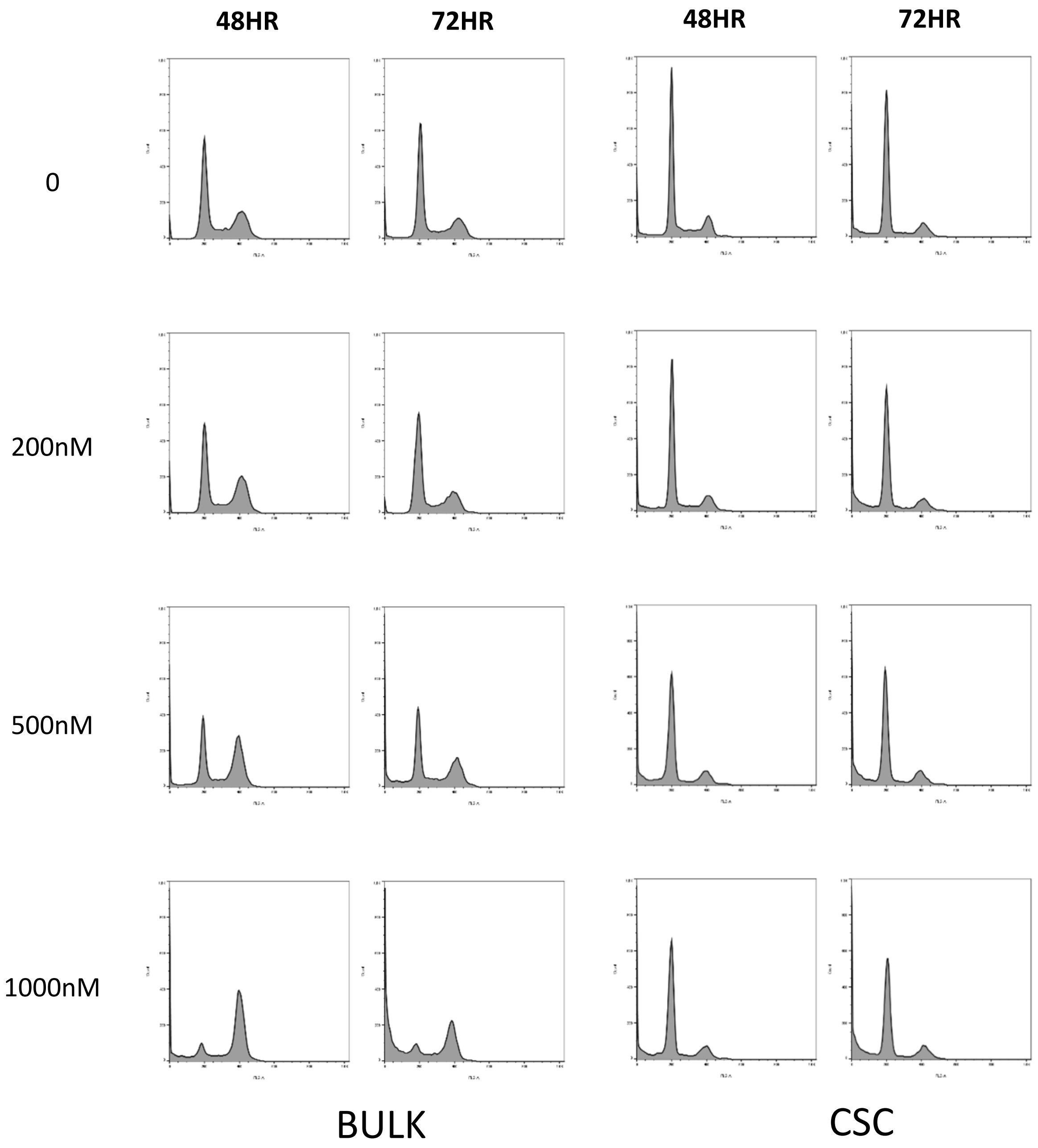

Supplementary Figure 2

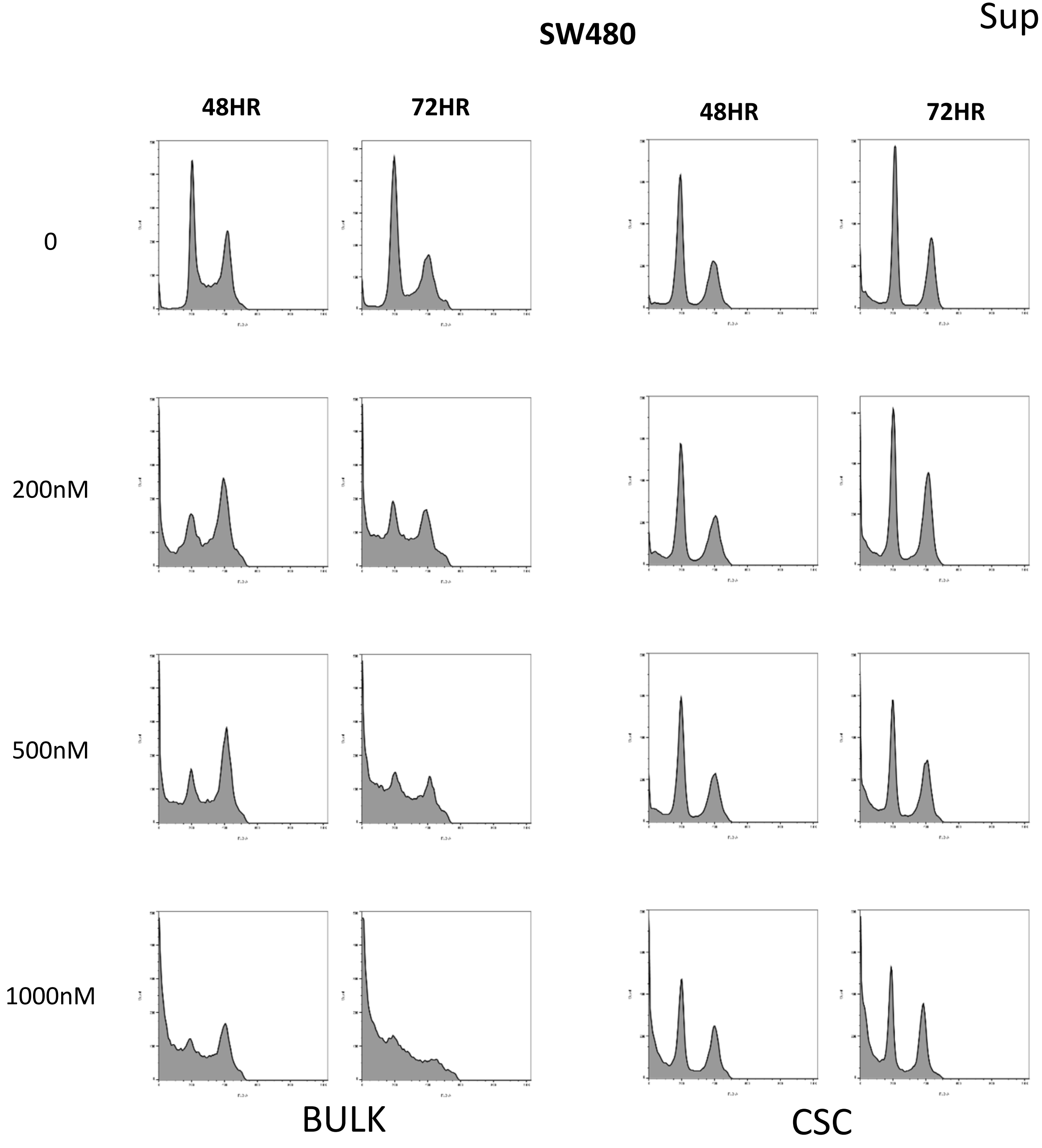

Supplementary Figure 3

SW480, BCC

16HR

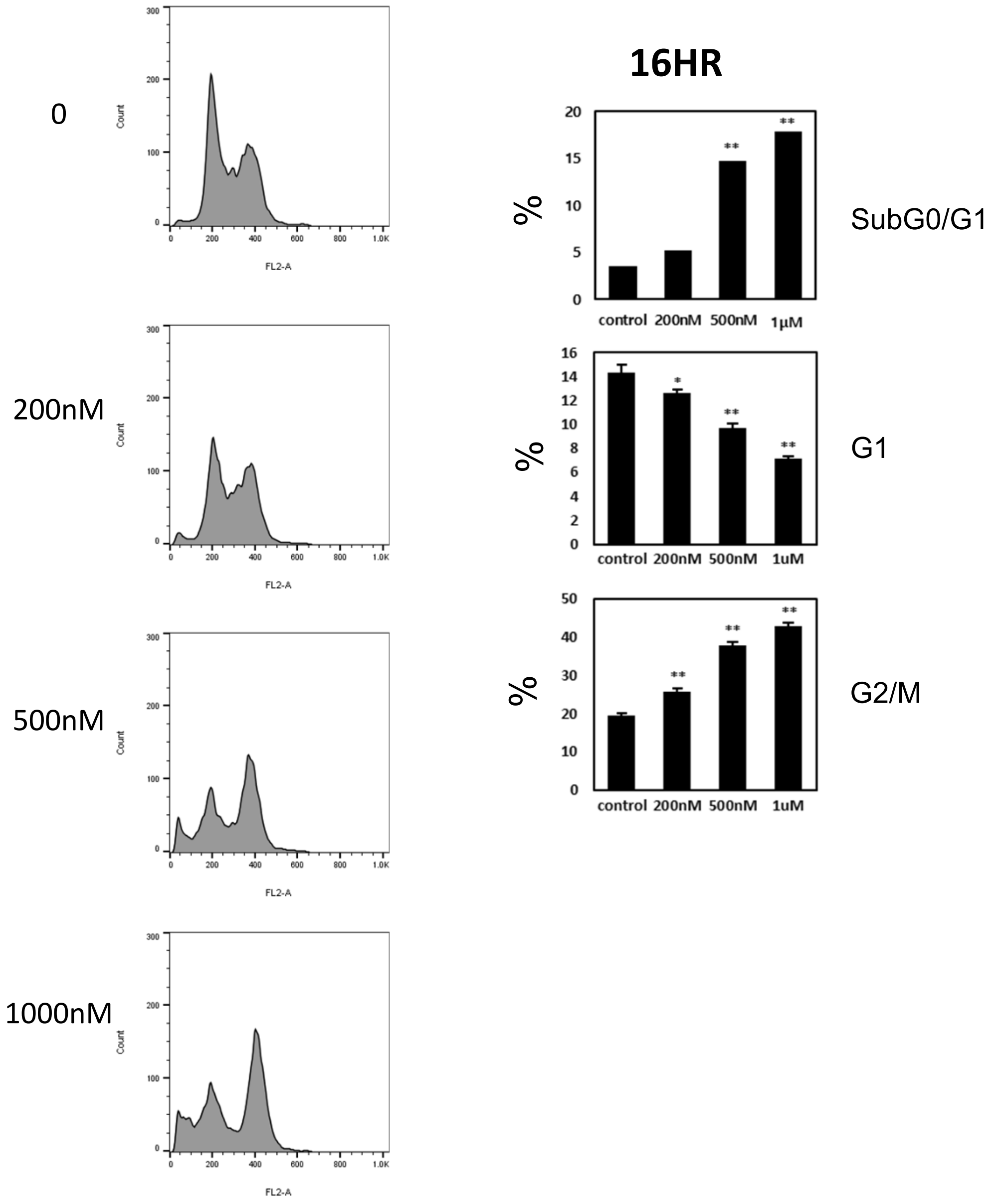

Supplementary Figure 4

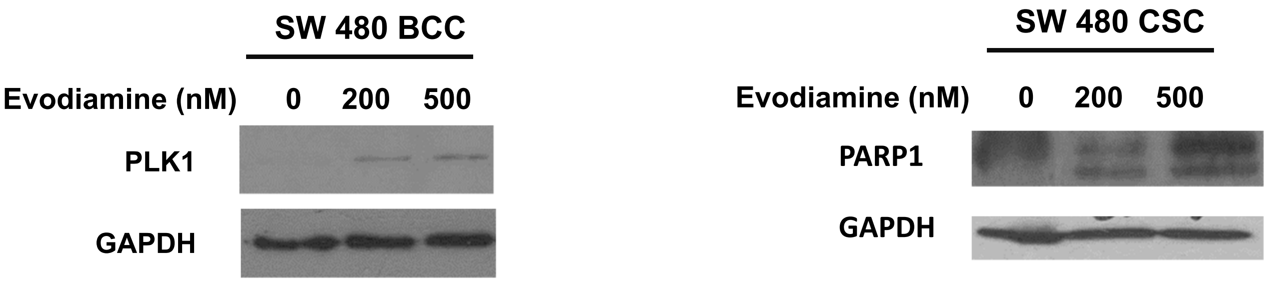

Supplementary Figure 5

SW480 cells 48hrs

<increase>

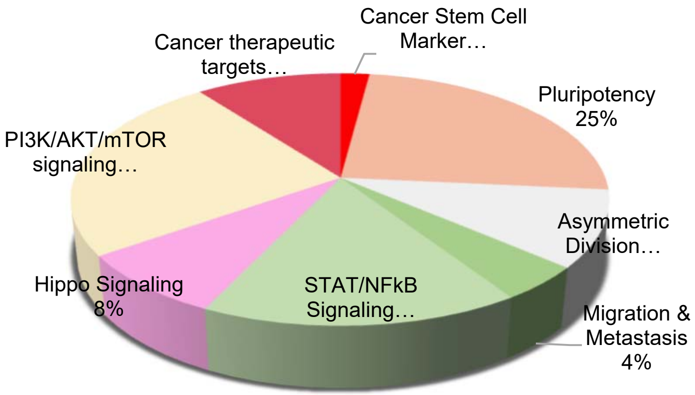

<decrease>

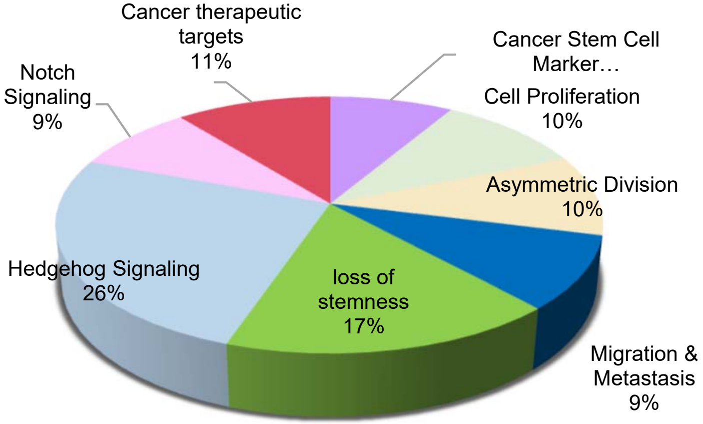

HT29 cells 72 hrs

<increase>

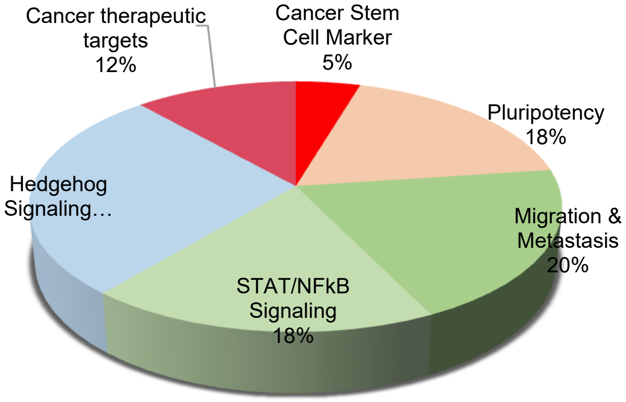

<decrease>

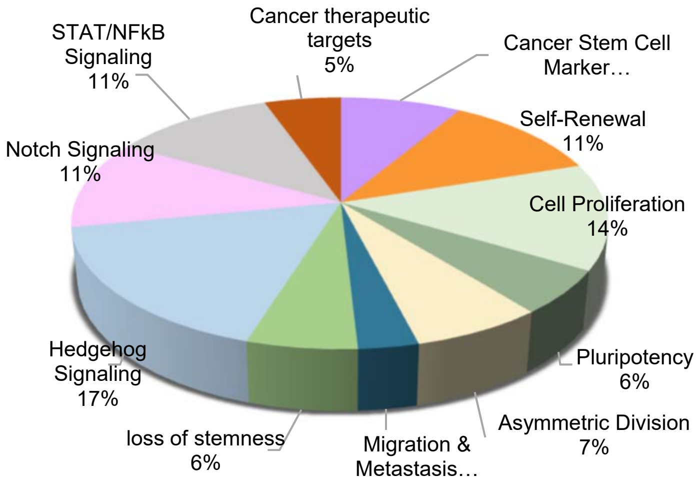

Supplementary Figure 6a

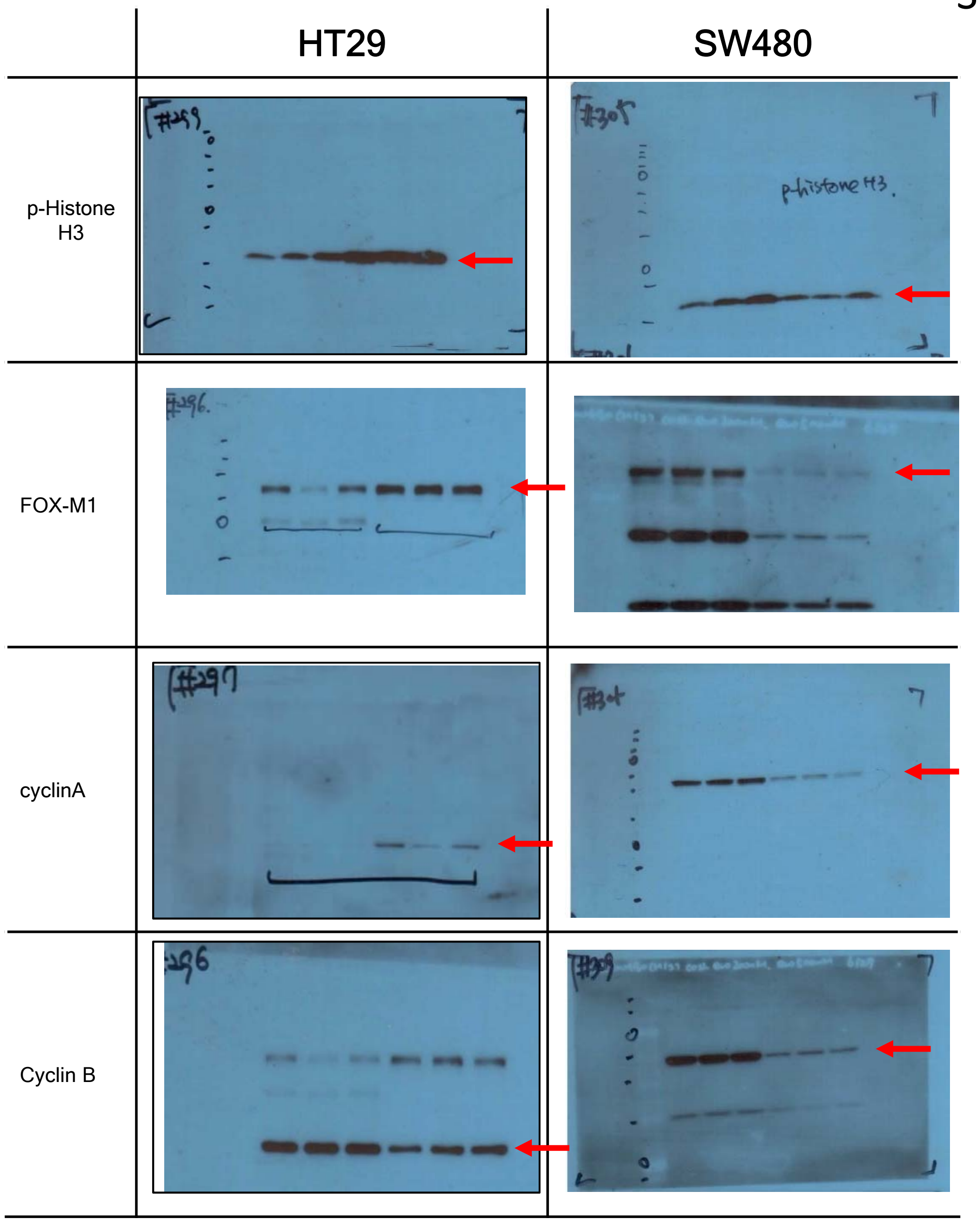

Supplementary Figure 6[illegible]

	HT29	SW480
Cyclin D1	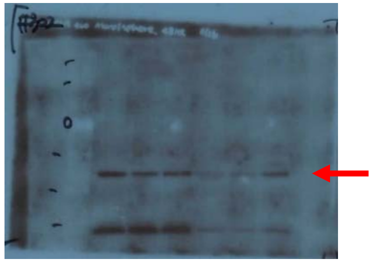	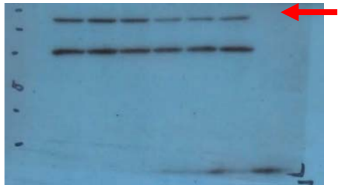
p21	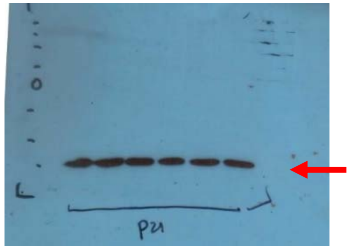	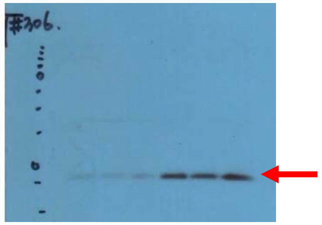
p53	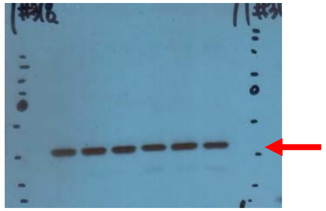	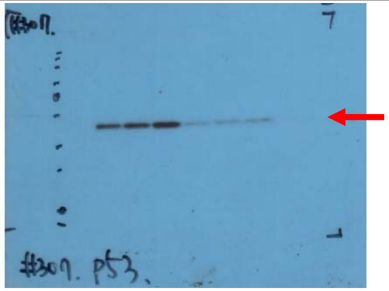
pRb(S807/811)	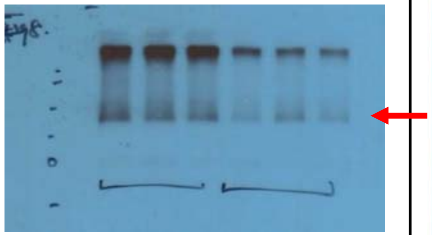	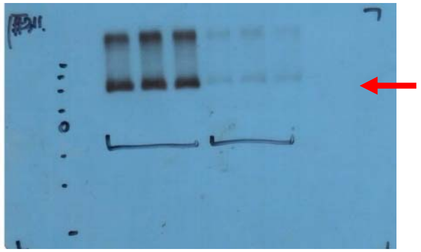

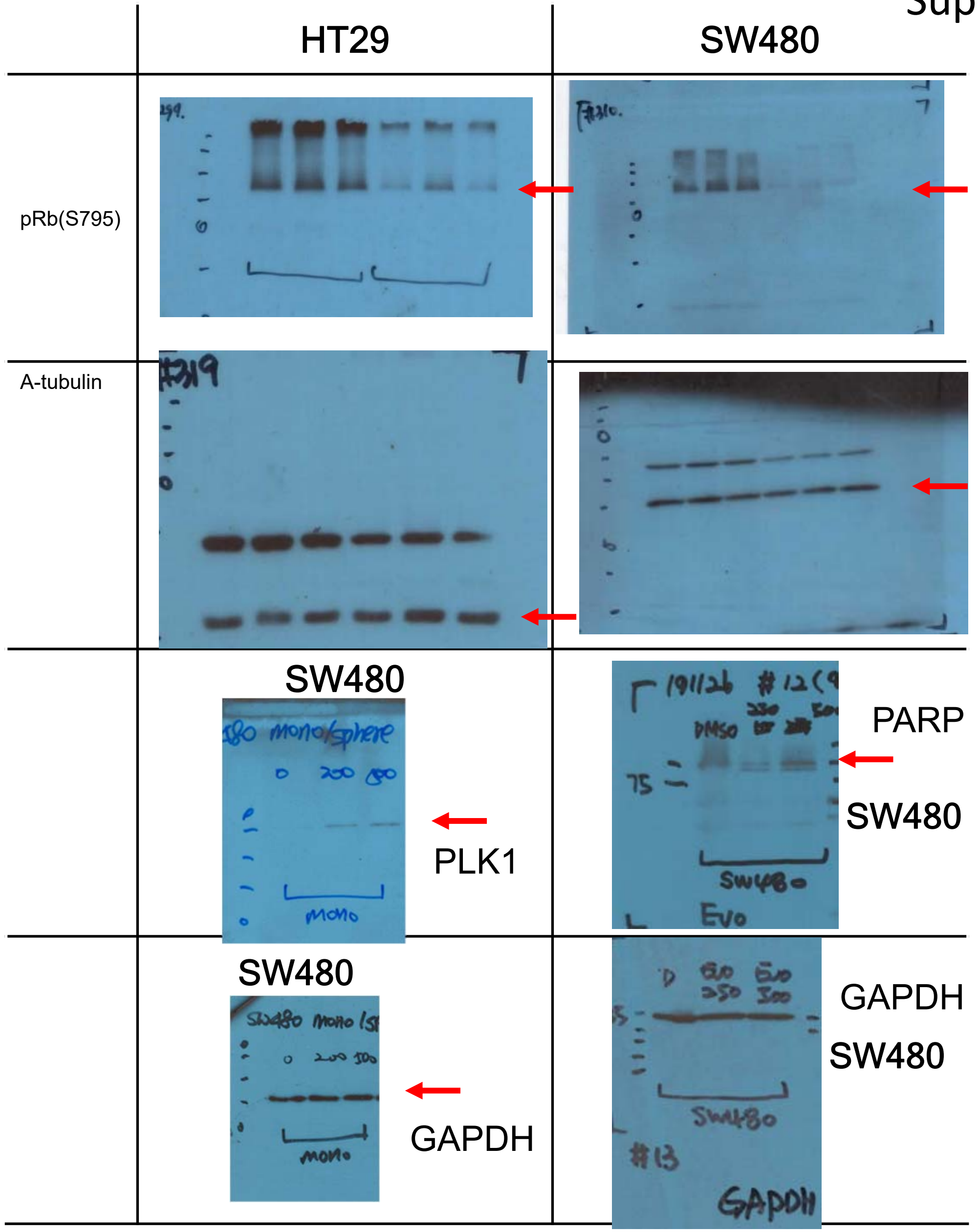

Supplementary Figure 6b

Supplementary Table 1. Realtime RT-PCR primer, used.

GENE	Sequence (5'→3')
CD133	F : TCA GTG AGA AAG TGG CAT CG<br>R : TGT TGT GAT GGG CTT GTC AT
FGFR3	F : GAG AAC AAG TTT GGC AGA AT<br>R : CGT CACTGT ACA CCT TGC AG
FZD9	F : TCC AGT ACG TGG AGA AGA GC<br>R : CAT GGA GAG GAA GAT GAT GG
GAPDH	F : TGC ACC ACC AAC TGC TTA GC<br>R : GGC ATG GAC TGT GGT CAT GAG
GLI1	F : CCC AAT CAC AAG TCA GGT TCC T<br>R : CCT ATG TGA AGC CCT ATT TGC C
HES1	F : GAG CAC AGA AAG TCA TCA AAG CCT ATC<br>R : GCC GGG AGC TAT CTT TCT TAA GTG C
HES5	F : TGG AGA AGG CCG ACA TCC T<br>R : GGC GAC GAA GGC TTT GC
L32	F : CAA CAT TGG TTA TGG AAG CAA CA;<br>R : TGA CGT TGT GGA CCA GGA ACT
LEF1	F : AGG AAC ATC CCC ACA CTG AC<br>R : AGG TCT TTT TGG CTC CTG CT
LRP5	(PPH02315F-200, Qiagen)
NOTCH1	F : CAA CAT CCA GGA CAA CAT GG<br>R : GGA CTT GCC CAG GTC ATC TA
SCD1	F : TGC CCA CCA CAA GTT TTC AG<br>R : CAT CAG CAA GCC AGG TTT GT
SMO	(PPH02222C-200, Qiagen)

Supplementary Table 2. Antibody list

Abs	Manufacture	Cat. #
**α-tubulin**	Santa Cruise	sc23948
**Cyclin A**	Cell signaling	#4656
**Cyclin B1**	Cell signaling	#4138
**Cyclin D1**	Cell signaling	#2978
**FoxM1**	Cell signaling	#5436
**P21**	Cell signaling	#2947
**P53**	Santa Cruise	sc129
**p-Histone H3**	Cell signaling	#3377
**pRB(S795)**	Cell signaling	#9301
**pRB(S807/S811)**	Cell signaling	#856
**Histone 3**	LifeSpan Bioscience	LS-C413067
**PLK-1**	Santa Cruise	sc55504
